# Fracture Criterion Calibration and Finite Element Simulation in SUS304 Stainless Steel

**DOI:** 10.3390/ma17235711

**Published:** 2024-11-22

**Authors:** Menglong Xing, Yangchao Liu, Jianwei Lu, Xiaomin Huang, Xinge Wang, Jianqi Xue, Fuming Zhang, Fengshan Du

**Affiliations:** 1 College of Mechanical Engineering, North China University of Science and Technology, Tangshan 063210, China; 2 Qing Gong College, North China University of Science and Technology, Tangshan 064000, China; 3 College of Automotive Engineering, Tangshan Polytechnic University, Tangshan 063299, China; 4 Cold Rolling Operation Department, Shougang Jingtang United Iron and Steel Co., Ltd., Tangshan 063200, China; 5 National Engineering Research Center for Equipment and Technology of Cold Strip Rolling, Yanshan University, Qinhuangdao 066004, China

**Keywords:** SUS304 stainless steel, ductile fracture criterion, finite element simulation, HYPELA2 subroutine

## Abstract

In order to calibrate the ductile fracture criterion in SUS304 stainless steel, four tensile samples were designed, their finite element models were established, and uniaxial tensile tests were carried out. The simulation results were compared with the tests. Due to the limitations of finite element software, only a few criteria can be solved in MENTAT of MSC.MARC 2013. In order to make the ductile fracture criteria universal, the HYPELA2 subroutine was used to write the ductile fracture criteria, and the three-point bending simulation was used to verify the ductile fracture criteria program. Finally, the calibration results of different fracture criteria are compared with the test results. The results show that the error between the finite element results of four samples and the test results is small, which verifies the feasibility of the finite element model. Based on the Oh criterion, the correctness of HYPELA2 subroutine development is verified by a three-point bending simulation. In the SH sample, the Freudenthal, C-L, and LeRoy criteria have a better prediction ability, and the prediction error of other criteria is relatively large. All the criteria have a strong prediction ability for the NT20 and CH05 samples.

## 1. Introduction

**Figure 1:**
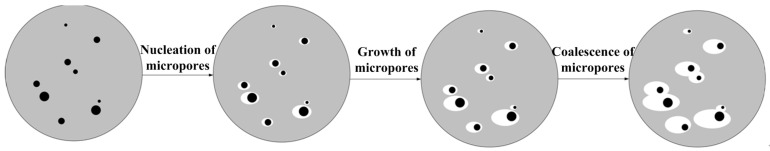
The process of crack formation.

Ductile fracture easily occurs when metal materials are subjected to large plastic deformation. Before the material breaks, due to the action of the load, the internal structure of the material will change, and the appearance of tiny holes, cracks, and other defects will gradually deteriorate the performance of the metal material; these tiny defects constitute damage, and when the damage accumulates to a certain extent, it will lead to the failure of the metal material fracture. The formation process of cracks mainly includes the nucleation, growth, and coalescence of the initial pores, as shown in Figure 1.

In related research on damage, it can be inferred from the interaction between fracture damage and plastic deformation that the ductile fracture criteria can generally be divided into two types: coupled and uncoupled. In the coupled damage model, scholars have considered the influence of damage evolution on the mechanical properties and deformation capacity of materials during plastic deformation. The uncoupled fracture criterion does not consider the interaction between damage and plastic deformation and generally uses macroscopic physical quantities as the criterion for damage occurrence [1]. For the coupled ductile fracture criterion, Gurson A. [2] proposed the GTN model in 1977, which introduces internal variables representing the volume fraction of micropores in the constitutive relationship and considers macroscopic hydrostatic stress to establish the yield function of porous materials. References [3,4,5,6,7] conducted a series of studies based on the GTN model. In 1958, Kachanov L. [8] proposed a model that represents continuous fracture variables through damage evolution equations and describes the local distribution of micro defects, known as the CDM model. References [9,10,11] made a series of revisions to the CDM model. Lemaitre J. [12] proposed a novel toughness fracture criterion based on continuous damage variables. Although the coupled fracture criterion is more in line with the actual process of damage development and material deformation, the constitutive relationship between damage and plasticity, simulation implementation, and parameter solving are relatively complex. In contrast, although the uncoupled fracture criterion ignores the constitutive relationship between damage and plasticity, its parameters are relatively few and the calibration process is relatively simple, meaning that it is widely used in engineering.

Uncoupled ductile fracture criteria are widely used in plastic forming processes such as deep drawing, roll bending, and spinning. Typical uncoupled ductile fracture criteria include the Cockcroft–Latham criterion, Rice–Tracey criterion, Brozzo criterion, Oh criterion, and Lou–Huh criterion. These criteria are constructed either in terms of the first principal stress, or the hydrostatic stress, or the equivalent stress, or a combination of several of these stresses, or the two parameters of stress triaxiality and Lode angle parameter as the influencing factors. Takuda H. [13] introduced the Oyane–Sato criterion into finite element simulation and successfully predicted the failure problem in the sheet metal reaming–forming process. Talebi-Ghadikolaee H. [14,15] used a variety of ductile fracture criteria to study the fracture behavior of AA6061-T6 aluminum alloy during roll bending. A variety of notched samples were used to calibrate the criteria, and the calibrated criteria were embedded in the finite element to achieve the prediction of fracture occurrence during roll bending. Based on 6005A aluminum alloy profiles, Liu C.G. [16] proposed a method for predicting roll bending fracture in aluminum alloy profiles considering anisotropy, and the results showed that the finite element model coupled with the anisotropic yield criterion and the continuous damage criterion was more accurate than the isotropic prediction results. Chen D. [17] used DIC technology to obtain the fracture strain of notched samples and calibrated the Lou–Huh criterion for fracture prediction during deep drawing. Based on the ductile damage theory of the Oyane criterion, Xia Q.X. [18] established a ductile fracture criterion that can accurately predict the ultimate shear spinning thinning rate by considering the influence of the average stress and maximum shear stress on the ductile damage process.

**Figure 2:**
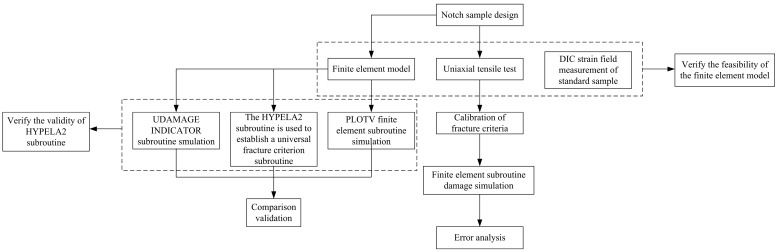
The technical flow chart.

Based on the above research, in this paper, a variety of notched tensile samples were designed, and unidirectional tensile tests were carried out. DIC technology was used to measure the strain field of standard samples. The finite element simulation model of notch sample was performed, and the experimental results were compared with the simulation results. The feasibility of the finite element model was verified by the DIC analysis results and the tensile test results of the notch sample. The finite element subroutine of the fracture criterion with universal applicability was established, and its correctness verified by the three-point bending method. In this paper, different fracture criteria are used to evaluate different tensile samples, and error analysis is carried out to match the appropriate fracture criteria for different samples. The technical flow chart for this paper is shown in Figure 2.

## 2. Relevant Parameters and Criteria of Ductile Fracture

With the ductile fracture criterion, stress triaxiality is used to express the influence on ductile fracture. With the development of ductile fracture criteria, Lode angle parameters are gradually introduced into ductile damage, with which the stress state of materials can be expressed by stress triaxiality and Lode angle parameters.

Among the parameters related to the ductile fracture criterion, the hydrostatic stress is

(1) $$\sigma_{m}=\frac{1}{3}(\sigma_{x}+\sigma_{y}+\sigma_{z})$$

where $\sigma_{x}$, $\sigma_{y}$, and $\sigma_{z}$ are the normal stresses in the *x*, *y*, and *z* directions, respectively. $\sigma_{m}$ is the hydrostatic stress.

The second invariant of the stress partial tensor is

(2) $$J_{2}=\frac{1}{6}[(\sigma_{x}-\sigma_{y}{)}^{2}+(\sigma_{y}-\sigma_{z}{)}^{2}+(\sigma_{z}-\sigma_{x}{)}^{2}]+\tau_{xy}^{2}+\tau_{yz}^{2}+\tau_{zx}^{2}$$

where $\tau_{xy}$, $\tau_{yz}$, and $\tau_{zx}$ are shear stresses on the *xy*, *yz*, and *zx* planes, respectively.

The third invariant of the stress partial tensor is

(3) $$J_{3}=\det(\sigma -\sigma_{m}I)$$

where det is the value of the determinant and I is the identity matrix.

The deviational stress state and shear effect also significantly affect the fracture behavior of metals. The Lode angle parameter is a key parameter to describe the shear effect, and it has an important effect on the deformation of micropores near the fracture, especially under the condition of low stress triaxiality. The Lode angle parameter is expressed as

(4) $$\bar{\theta }=1-\frac{2}{\pi }\arccos[\frac{3\sqrt{3}}{2}\frac{J_{3}}{(J_{2}{)}^{\frac{3}{2}}}](-1\leq \bar{\theta }\leq 1)$$

The equivalent stress is

(5) $$\sigma_{e}=\sqrt{\frac{1}{2}[(\sigma_{x}-\sigma_{y}{)}^{2}+(\sigma_{y}-\sigma_{z}{)}^{2}+(\sigma_{z}-\sigma_{x}{)}^{2}]+3(\tau_{xy}^{2}+\tau_{yz}^{2}+\tau_{zx}^{2})}$$

Stress triaxiality is a key index used to evaluate fracture damage behavior in metal materials. In terms of microscopic performance, under the condition of high stress triaxiality, the fracture form of the metal is usually tensile fracture, which is manifested as a significant increase in, and polymerization of, the holes after nucleation under the condition of tensile stress.

In the case of low stress triaxiality, the fracture mode of the metal is mainly shear fracture, which is manifested in the holes gradually becoming elongated along the direction of the maximum shear stress and the shape changing significantly but the volume changing little.

The stress triaxiality is

(6) $$\eta =\frac{\sigma_{m}}{\sigma_{e}}$$

where $\sigma_{e}$ is the equivalent stress (MPa).

Based on the improved Haigh–Westergaard coordinates [19], the trigonometric function related to the Lode angle parameter is

(7) $$f_{1}[\bar{\theta }]=\frac{2}{3}\cos[\frac{\pi }{6}(1-\bar{\theta })]$$

(8) $$f_{2}[\bar{\theta }]=\frac{2}{3}\cos[\frac{\pi }{6}(3+\bar{\theta })]$$

(9) $$f_{3}[\bar{\theta }]=-\frac{2}{3}\cos[\frac{\pi }{6}(1+\bar{\theta })]$$

The first principal stress, the second principal stress, and the third principal stress are converted to

(10) $$\sigma_{1}=\sigma_{e}(\eta +f_{1})$$

(11) $$\sigma_{2}=\sigma_{e}(\eta +f_{2})$$

(12) $$\sigma_{3}=\sigma_{e}(\eta +f_{3})$$

As an empirical model, the uncoupled ductile fracture criterion usually adopts the function related to the stress state to express the fracture damage behavior caused by the accumulation of plastic deformation, so it is called the fracture damage criterion based on the stress state, and the generalized expression is as follows

(13) $$\int_{0}^{{\bar{\varepsilon }}_{f}^{p}}f(\sigma )d{\bar{\varepsilon }}^{p}=C_{s}$$

where f(σ) is a function related to the stress state; ${\bar{\varepsilon }}_{f}^{p}$ is the critical equivalent fracture strain; and $C_{s}$ is the dimensionless damage threshold. When the integral term on the left side of the equation reaches $C_{s}$, the material is considered to have begun to undergo ductile damage, and ${\bar{\varepsilon }}_{f}^{p}$ is the corresponding equivalent plastic strain in this state. As can be seen from Equation (13), the specific form of the fracture criterion expression depends entirely on the form of f(σ). Typical ductile fracture criteria (Freudenthal [20], C-L [21], Oh [22], R-T [23], Brozzo [24], McC [25], LeRoy [26], L-H [27], H-C [28]) are shown in Table 1.

## 3. Calibration of Fracture Criteria

### 3.1. Uniaxial Tensile Tests and Finite Element Modeling

**Figure 3:**
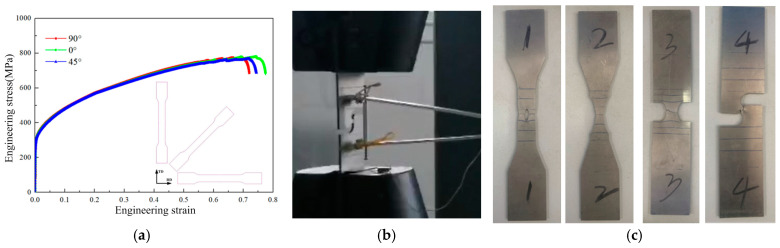
Uniaxial tensile test results for samples: (**a**) SUS304 DB sample uniaxial tensile test results. (**b**) Uniaxial tensile test procedure. (**c**) Uniaxial tensile test results for notched samples.

In order to analyze the mechanical behavior of tensile samples under different stress states from plastic deformation to fracture, uniaxial tensile tests were carried out on the DB sample, CH05 sample, NT20 sample, PST sample, and SH sample. The test is carried out on an electronic universal test machine, which drives the sample until it breaks. All tests were performed at room temperature. DB samples with rolling directions of 0°, 45°, and 90° were cut for the uniaxial tensile test, and the stress–strain curves are shown in Figure 3a. It can be seen that SUS304 stainless steel does not show an obvious yield platform, and the difference in the strain hardening curve in all directions is not significant; it can be assumed that the plate conforms to isotropy. The thickness of the tensile sample is 3 mm, the tensile rate is 3 mm/min, and the length of the standard distance segment is 50 mm. The process and results of the tensile test are shown in Figure 3b and Figure 3c, respectively.

**Figure 4:**
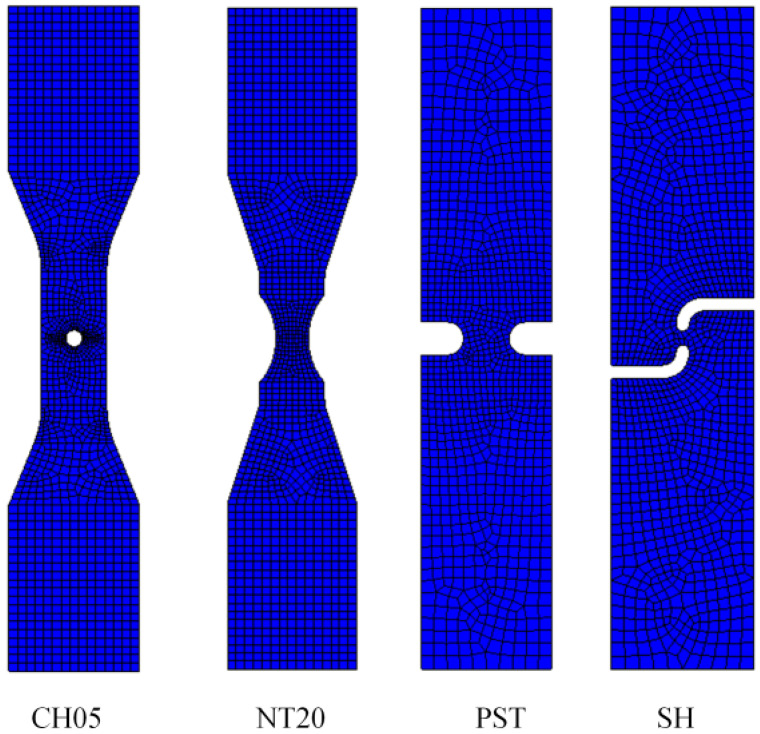
Finite element model of four types of samples.

Finite element models of four kinds of samples were established. Element No. 7 was used for mesh division; local deformation areas were refined for mesh. The method of mesh refinement means that the closer the deformation area is, the greater the number of seed points corresponding to it. The bottom of the sample was fully constrained. The model was established as shown in Figure 4. The material model adopts the SWIFT model, the fitting formula is shown in (14), and the correlation coefficient is 0.99996, indicating a good fitting effect.

(14) $$\sigma =1840.41\cdot {\left(\varepsilon_{p}+0.1665\right)}^{0.947}$$

**Figure 5:**
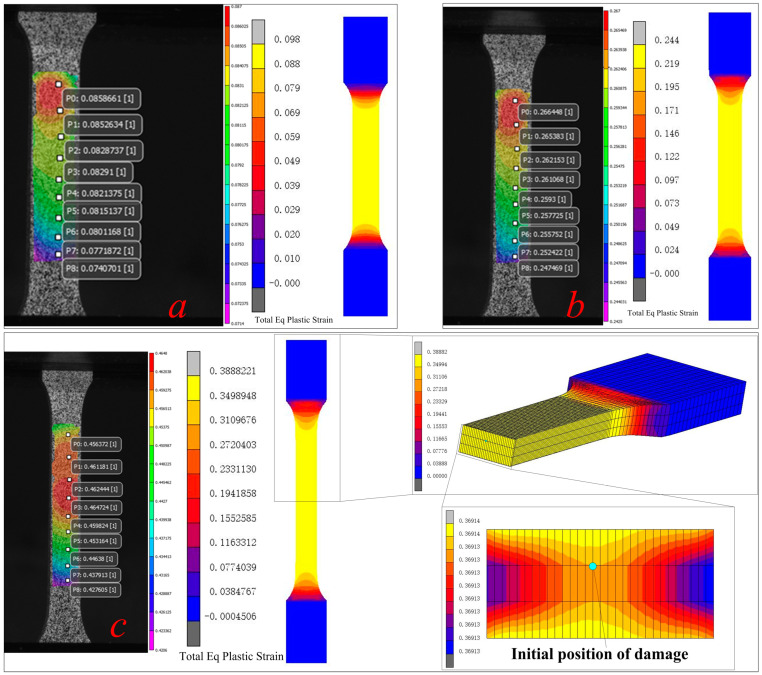
Comparison of DIC test results with simulation results for the DB sample. (**a**) DB sample deformation in the initial stage (**b**) DB sample deformation in the early stage (**c**) DB sample deformation in the middle stage.

In order to better verify the feasibility of the finite element model, DIC technology was used to measure the strain field of DB samples, and the simulation results were compared with DIC results, as shown in Figure 5. It can be seen that with the progression of the uniaxial tensile test, the error of equivalent plastic strain is gradually increased, but it is still within the acceptable range, which preliminarily verifies the feasibility of the simulation method.

**Figure 6:**
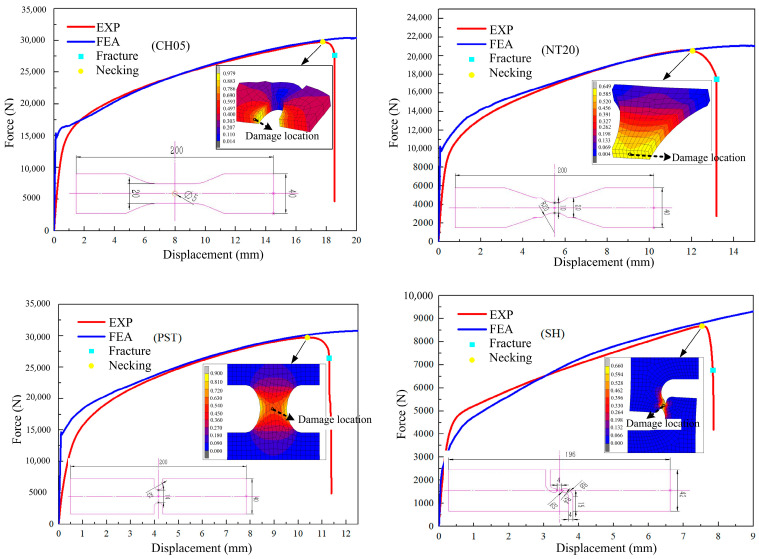
The simulated force curve of four samples.

The simulation force path curve of the sample was calculated with the finite element simulation results, and the simulation force path curve was compared with the test curve, as shown in Figure 6. It can be seen that the error of the shear sample was larger among the four samples, while the error of the other three samples was smaller, but all of them were within the acceptable range, which verified the feasibility of the finite element prediction method.

**Figure 7:**
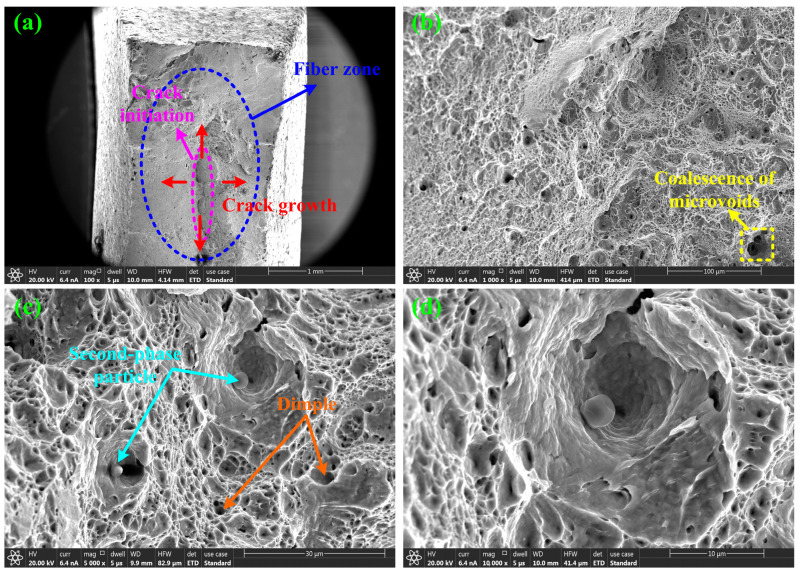
DB sample fracture morphology of SUS304 stainless steel. (**a**) 100× (**b**) 1000× (**c**) 5000× (**d**) 10,000×.

**Figure 8:**
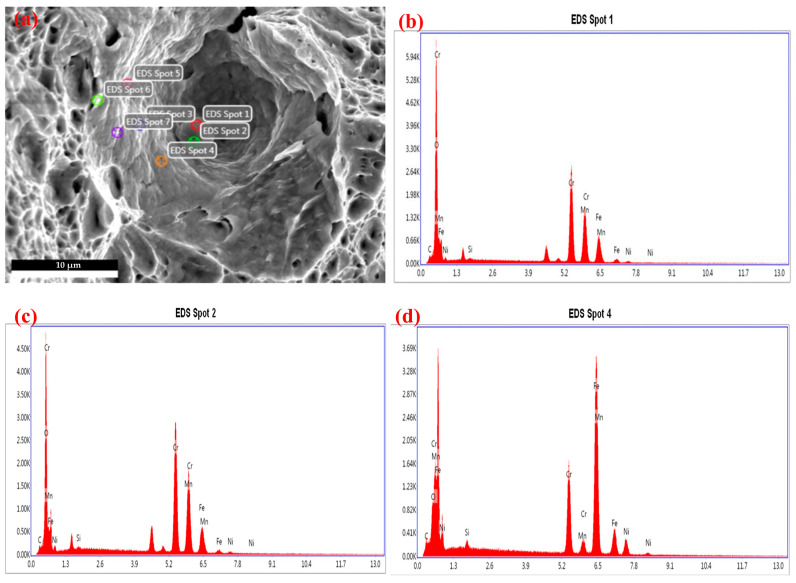
EDS analysis of DB sample fracture. (**a**) 10,000× EDS analysis (**b**) EDS spot 1 (**c**) EDS spot 2 (**d**) EDS spot 4.

The fracture of the DB sample was observed using the scanning electron microscope. The surface morphology of the fracture is shown in Figure 7. From Figure 7c, it can be seen that there are a large number of dimples on the surface of the fracture, and the dimples are deep. It can be seen from Figure 7a that the fiber region of the fracture is large, and the location of the crack source is determined to be in the middle region of the fracture. In addition, it can also be seen that the coalescence of microvoids is obvious in Figure 7b, and the second-phase particles are obvious in Figure 7d. According to the above characteristics, the fracture is determined to be a ductile fracture. Point scanning was performed on Figure 7d, and the EDS results are shown in Figure 8. It can be seen that Cr has a high content of chemical components. Although the fracture can provide a degree of support for the determination of the fracture location, the key problem of fracture criterion calibration is the relationship between the formation, growth, coalescence of micropores, and force curve, which has not yet been determined. At the same time, the ductile fracture criterion itself does not emphasize the microscopic process of the evolution and development of pores but pays more attention to the macroscopic mechanical performance initiated by the evolution and development of pores.

### 3.2. Damage Parameter Simulation Based on MSC.MARC Subroutine

**Figure 9:**
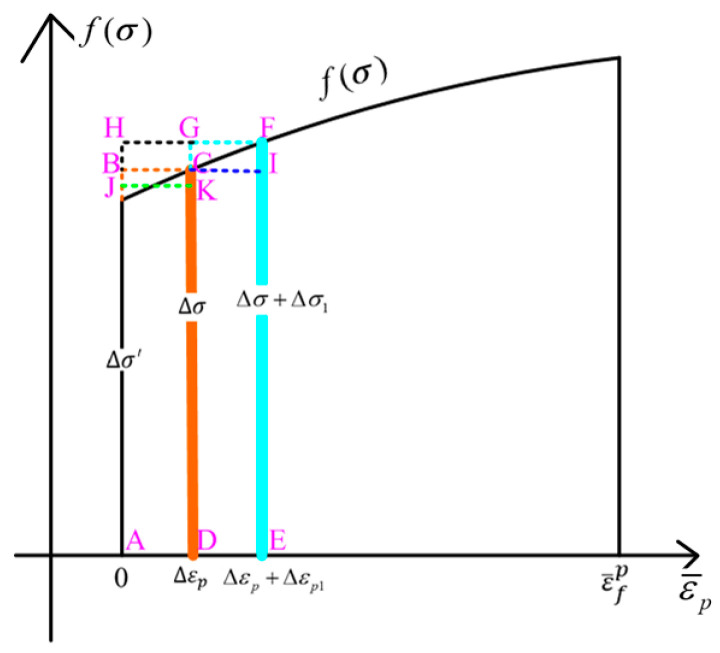
Diagram of the result of a subroutine calculation.

The damage value during the uniaxial tensile test simulation can be output by the UDAMAGE_INDICATOR subroutine or PLOTV subroutine in MSC.MARC 2013. The stress triaxiality and Lode angle parameters are output by the PLOTV subroutine. The calculation of the two user subroutines will produce certain deviations due to different iteration methods, which are described as follows:

When the PLOTV subroutine is used to calculate the current stress state function value, the damage value of the first step is 0, in the second step is $\Delta \sigma \cdot \Delta \varepsilon_{p}$, and in the third step is $\left(\Delta \sigma +\Delta \sigma_{1})\cdot (\Delta \varepsilon_{p}+\Delta \varepsilon_{p1}\right)$. When the PLOTV subroutine is used to calculate the average stress state function value, the damage value in the first step is 0, in the second step is $\frac{\left(\Delta \sigma^{\prime }+\Delta \sigma \right)}{2}\Delta \varepsilon_{p}$, and in the third step is $\frac{\left(\Delta \sigma^{\prime }+\Delta \sigma +\Delta \sigma_{1}\right)}{2}(\Delta \varepsilon_{p}+\Delta \varepsilon_{p1})$. When the UDAMAGE_INDICATOR subroutine is used, the damage value of the first step is 0, and in the second step, it is $0+\Delta \sigma \cdot \Delta \varepsilon_{p}$. In the third step, it is $0+\Delta \sigma \cdot \Delta \varepsilon_{p}+(\Delta \sigma +\Delta \sigma_{1})\cdot \Delta \varepsilon_{p1}$. It can be seen that the difference in the three calculation methods leads to a certain deviation in the damage value. The specific geometric description is shown in Figure 9.

It can be seen that when the PLOTV subroutine is used to calculate the current stress state function value, the damage value of the first step is 0, the damage value of the second step is the area of the rectangular ABCD, and the damage value of the third step is the area of the rectangular AEFH. When the PLOTV subroutine is used to calculate the average stress state function value, the damage value of the first step is 0, the damage value of the second step is the area of the rectangular AJKD, and the damage value of the third step is the area of the rectangular ABIE. When the UDAMAGE_INDICATOR subroutine is used, the damage value of the first step is 0, the damage value of the second step is the area of the rectangular ABCD, and the damage value of the third step is the sum of the area of the rectangular ABCD and the DEFG area. It can be seen that when the PLOTV subroutine is used to calculate the corresponding damage value according to the current stress state function value, the calculation result is relatively conservative, but the calculation efficiency is relatively high. When the PLOTV subroutine is used to calculate the average stress state function and the UDAMAGE_INDICATOR subroutine is used, the calculation results are relatively accurate, but the calculation efficiency is low.

**Figure 10:**
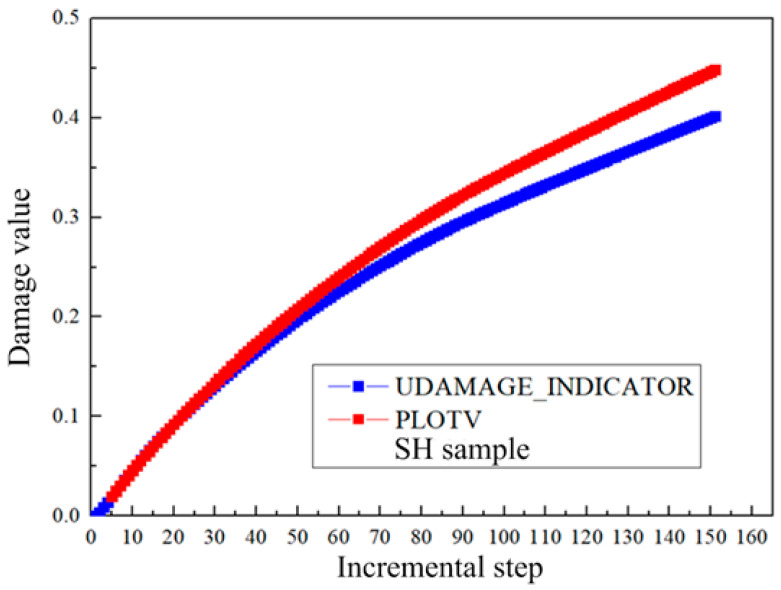
Comparison of the calculated damage values.

**Figure 11:**
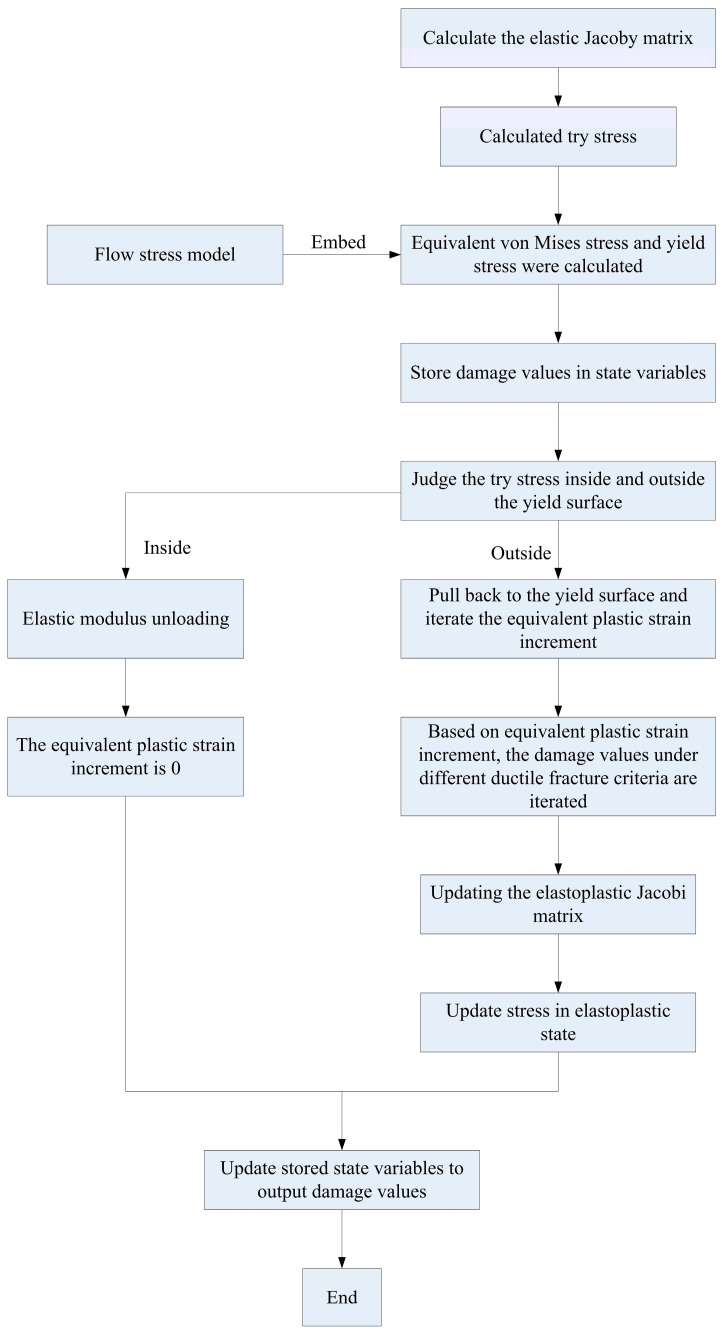
Damage calculation procedure of the HYPELA2 subroutine.

Taking the PLOTV subroutine and the UDAMAGE_INDICATOR subroutine calculated by the current stress state function value as an example, the shear sample is verified based on the Oh criterion, as shown in Figure 10. It can be seen that the calculation result of the PLOTV subroutine according to the current stress state function value is larger than that of the UDAMAGE_INDICATOR subroutine. However, only three damage criteria in MSC.MARC can use the UDAMAGE_INDICATOR damage indicator subroutine for damage value prediction. For other criteria, the limitations are relatively large. For the more accurate implementation of other damage criteria, the HYPELA2 subroutine can be used for iterative calculation. Figure 11 shows the calculation process. The damage calculation user subroutine calculation theory is as follows:

According to the subsequent yield criterion, the equivalent stress and yield stress in step n + 1 satisfy the following conditions:

(15) $$F=\bar{\sigma }-\sigma_{s}=0$$

Step n + 1 equivalent stress:

(16) $$\sigma_{y}={\bar{\sigma }}_{tr}-3G\Delta \bar{\varepsilon }$$

For linear flow models,

(17) $$\sigma_{y}=\sigma_{0}+H\varepsilon_{p}$$

For nonlinear flow models,

(18) $$\sigma_{y}=\varphi (\varepsilon_{p})$$

For nonlinear models, the Newton–Raphson method is used for an iterative solution:

(19) $$d\Delta \bar{\varepsilon }=\frac{\bar{\sigma }-3G\Delta \bar{\varepsilon }-\sigma_{y}}{3G+H^{\prime }}$$

The elastic–plastic Jacobi matrix is updated, as follows:

(20) $$D_{ijkl}^{eq}=\lambda \delta_{ij}\delta_{kl}+2G(\delta_{ik}\delta_{jl}+\delta_{il}\delta_{jk})-\frac{9G^{2}}{{{\bar{\sigma }}_{s}}^{2}(H^{\prime }+3G)}S_{ij}S_{kl}$$

where F is the yield surface; $\bar{\sigma }$ is the equivalent stress; $\sigma_{s}$ is the yield stress; ${\bar{\sigma }}_{tr}$ is the equivalent try stress; *G* is the shear modulus; $\Delta \bar{\varepsilon }$ is the equivalent plastic strain increment; $\sigma_{0}$ is the initial yield limit; *H* is the enhancement coefficient of the linear flow model; *H^’^* is the enhancement coefficient of the nonlinear flow model; $\varepsilon_{p}$ is the plastic strain; $D_{ijkl}^{eq}$ is the elastic–plastic Jacobi matrix; λ is the Lame coefficient; δ is the K symbol; ${\bar{\sigma }}_{s}$ is the equivalent von Mises stress; and *S* is the deviatoric stress.

**Figure 12:**
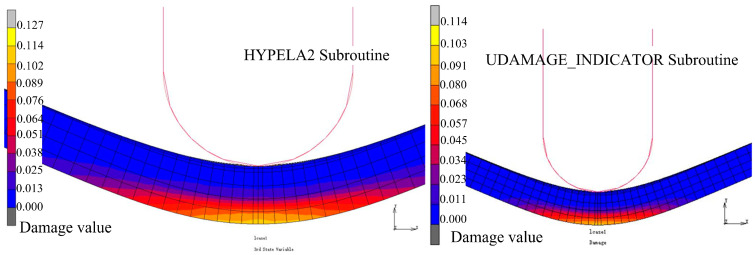
Comparison of the results of two subroutines.

**Figure 13:**
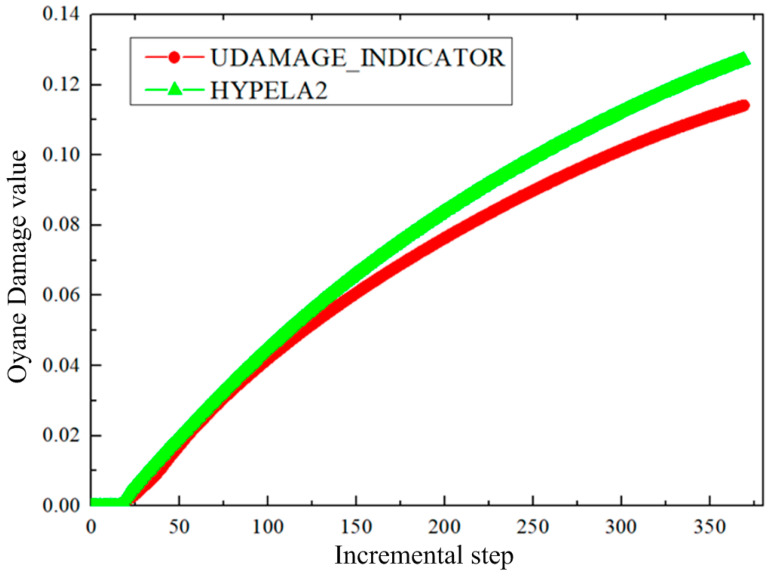
Change in damage value with incremental steps in two user subroutines.

When iterating the equivalent plastic strain increment, it is necessary to calculate the current damage accumulation according to the strain increment and store the damage accumulation in the state variable to facilitate the final result output. Based on the Oyane criterion, taking three-point bending forming as an example, the effects of the HYPELA2 subroutine and the UDAMAGE_INDICATOR subroutine on forming results were analyzed. When the material parameter B=0.5 in the Oyane criterion and incremental step size is 369 steps, the damage value distribution results of the two subroutines are shown in Figure 12. Taking the position of the center node of the outer layer of the sheet as the research objective, the change in damage value with the increment step is extracted, as shown in Figure 13. It can be seen that the distribution results for the damage value between the two are similar. The maximum value calculated by the HYPELA2 subroutine is larger than that calculated by the UDAMAGE_INDICATOR subroutine, but it is within the acceptable range. The feasibility of HYPELA2 subroutine calculation is verified.

Based on the above analysis, it can be seen that in order to improve efficiency, the PLOTV subroutine calculated according to the current stress state function value can be used for damage calculation during process parameter screening in engineering. The UDAMAGE_INDICATOR subroutine or the HYPELA2 subroutine can be used for damage prediction in the calculation, which requires high damage accuracy. At the same time, it can be seen from Figure 9 that the curve of the stress state function f(σ) changing with the equivalent plastic strain has an important influence on the calculation results produced by different calculation methods.

**Figure 14:**
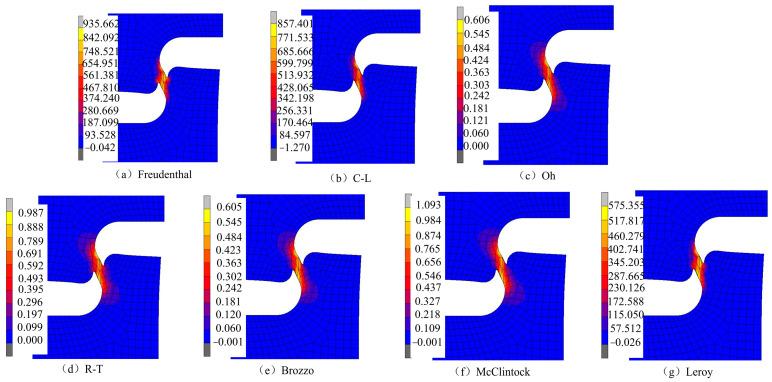
Damage prediction for the SH sample under different criteria.

**Figure 15:**
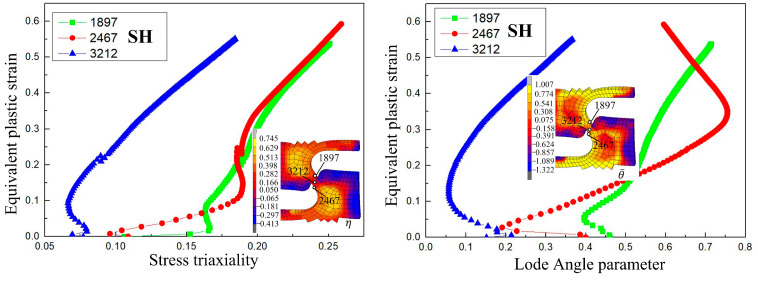
Stress triaxiality and Lode angle parameter change with equivalent plastic strain at different nodes for the SH sample.

The simulation results for the damage values in the SH sample under different ductile fracture criteria are shown in Figure 14. It can be seen that the distribution of damage values of Oh, R-T, Brozzo, and McC is similar, and the maximum damage values all appear at the position of the shear zone concave. The damage values for Freudenthal, C-L, and Leroy were similar, and the maximum damage values appeared in the middle and at the middle edge of the shear zone. According to the literature [29], the damage initiation point in the shear test starts at the location where the maximum plastic strain of the material occurs. Including the equivalent plastic strain distribution of the SH sample shown in Figure 6, it can be seen that the maximum strain occurs in the central and edge regions of the shear band area. According to Figure 15, the equivalent plastic strain of the SH sample under different stress triaxiality can be seen, which shows that although the stress triaxiality at the edge of the central region is larger than that in the central region, which is beneficial for crack initiation, the stress state in the central region is the closest to pure shear at this time. Among the fracture criteria selected, only the Freudenthal, C-L, and Leroy criteria can accurately predict the location of crack initiation in a pure shear fracture.

**Figure 16:**
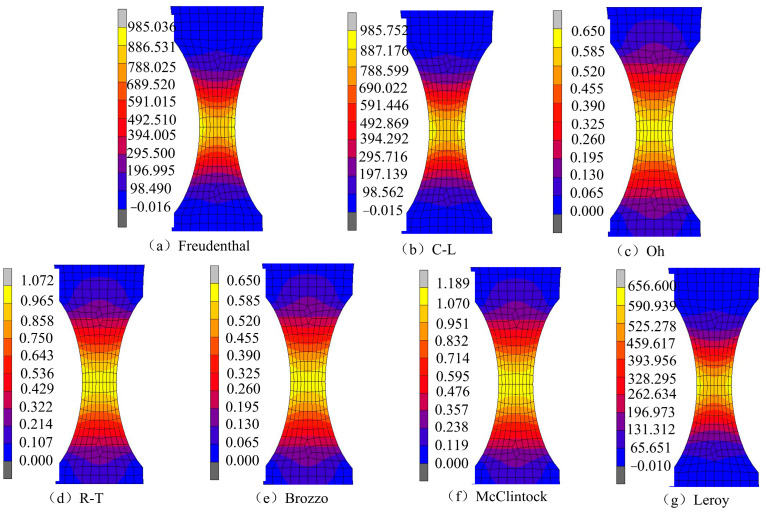
Damage prediction in the NT20 sample under different criteria.

**Figure 17:**
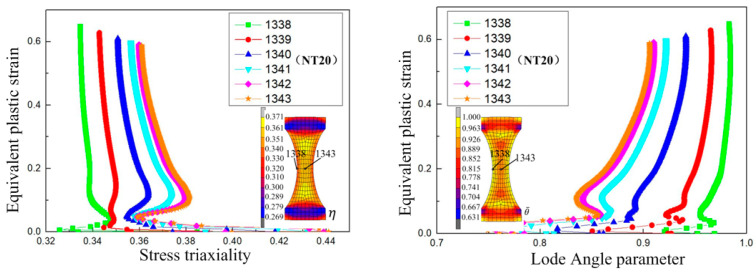
Stress triaxiality and Lode angle parameter change with equivalent plastic strain at different nodes in the NT20 sample.

Figure 16 shows the damage value distribution for the NT20 sample under different ductile fracture criteria. It can be seen that the maximum damage value in each criterion appears in the region with the smallest cross-section area of the sample. According to the equivalent plastic strain distribution of the NT20 sample in Figure 6, the maximum strain appears at the center point of the minimum section. Combined with the change in equivalent plastic strain with the stress triaxiality in Figure 17, it can be seen that the stress triaxiality at the center point of the minimum section is greater than that at other parts of the section, and the change law is significant. Higher stress triaxiality is conducive to the growth of holes and cracks, so the maximum equivalent plastic strain point (central position point) in the minimum necking section is taken as the key point of crack initiation. All of the selected ductile fracture criteria can accurately predict the initiation location of cracks in the NT20 sample.

**Figure 18:**
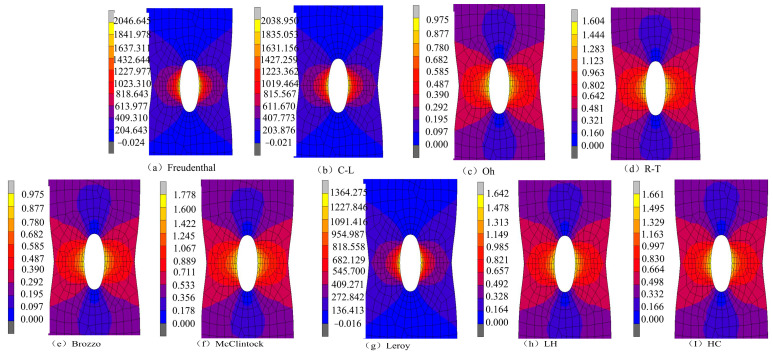
Damage prediction for theCH05 sample under different criteria.

**Figure 19:**
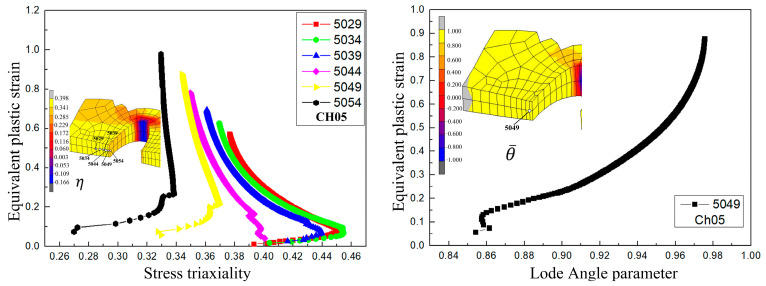
Stress triaxiality and Lode angle parameter change with equivalent plastic strain at different nodes for the CH05 sample.

Figure 18 shows the damage value distribution for the CH05 sample under different ductile fracture criteria. It can be seen that the maximum damage value predicted by the Freudenthal criterion and Leroy criterion is located on the inner ring surface of the central hole, while the other criteria are a certain distance from the inner ring surface. Combined with the equivalent plastic strain distribution of the CH05 in Figure 6, it can be seen that the maximum strain occurs at the center of the circumferential surface. According to the variation in the equivalent plastic strain with stress triaxiality of the CH05 sample (Figure 19), material points with a certain distance from the surface of the inner ring have higher stress triaxiality than those at the center of the circumferential surface, which is conducive to the expansion of the hole. Therefore, material points a certain distance from the surface of the inner ring are taken as the key points for crack initiation [30].

**Figure 20:**
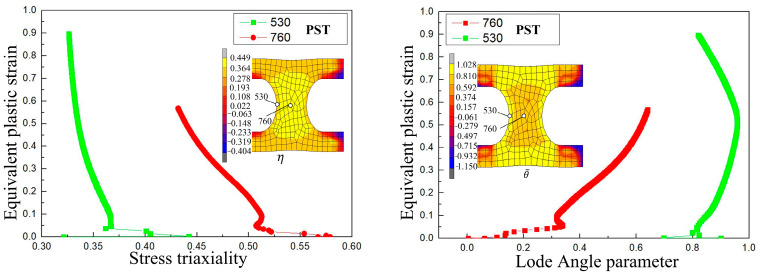
Stress triaxiality and Lode angle parameter change with equivalent plastic strain at different nodes for the PST sample.

According to Figure 6, the maximum equivalent plastic strain of the PST sample appears at the edge of the minimum section, which is higher than that in the central area. Combined with the change in equivalent plastic strain with stress triaxiality in the PST sample (Figure 20), it can be seen that the stress triaxiality at the central node of the minimum section is higher than that at the central edge, so node No. 760 is selected as the key point of crack initiation.

## 4. Damage Criterion Calibration and Error Comparison

According to the simulation results, test data, and crack initiation location in Figure 6, the split point between the test curve and the simulated force path curve was taken as the start time for crack initiation [31], and different damage criteria were calibrated. The first seven criteria had fewer parameters and were easy to calibrate, and the PST tensile sample was used to calibrate the first seven criteria. The L-H and H-C criteria require at least three samples to complete the calibration. Based on the three samples PST, SH, and NT20, the mean stress triaxiality and mean Lode angle parameters are used to solve the problem, and the specific results are shown in Table 2.

Based on the calibration results of the fracture criteria in Table 2, the simulation result for the damage value is compared with the damage threshold. When the simulation result is greater than the damage threshold, damage is considered to have begun to occur. The incremental step of the fracture time in the simulation is extracted for comparison, and the error evaluation index is as follows:

(21) $$e=\frac{T_{f}-T_{e}}{T_{e}}$$

where *e* is the relative error; $T_{f}$ is the moment when the calibrated criterion begins to generate damage in increment the step; and $T_{e}$ is the incremental step time when damage occurs in the simulation results corresponding to the test.

**Figure 21:**
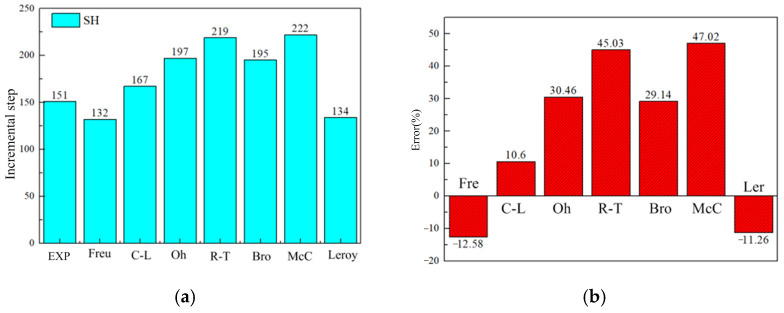
Damage initiation time for the SH sample under different criteria. (**a**) The corresponding incremental steps of damage appearing. (**b**) Damage error distribution.

Figure 21a shows the damage onset time for the SH sample under different criteria, and Figure 21b shows the relative error distribution of the SH sample. It can be seen that the Freudenthal, C-L, and LeRoy criteria have a strong prediction ability for the SH sample, while the errors of the other criteria are relatively large.

**Figure 22:**
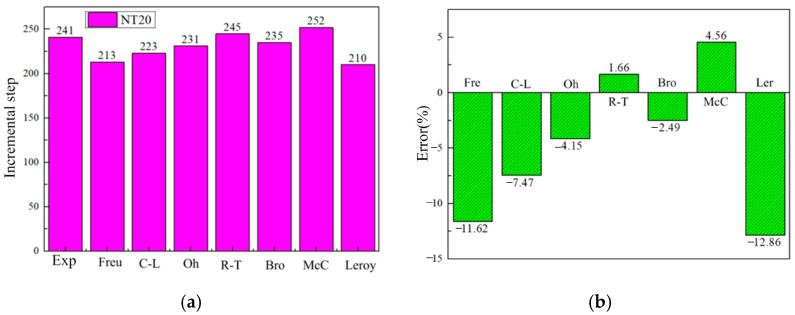
Damage initiation time for the NT20 sample under different criteria. (**a**) The corresponding incremental steps of damage appear. (**b**) Damage error distribution.

Figure 22 shows the time of damage occurrence and error distribution for the NT20 sample under different criteria. It can be seen that the error of all criteria is small, and all criteria can predict the fracture under high stress triaxiality.

**Figure 23:**
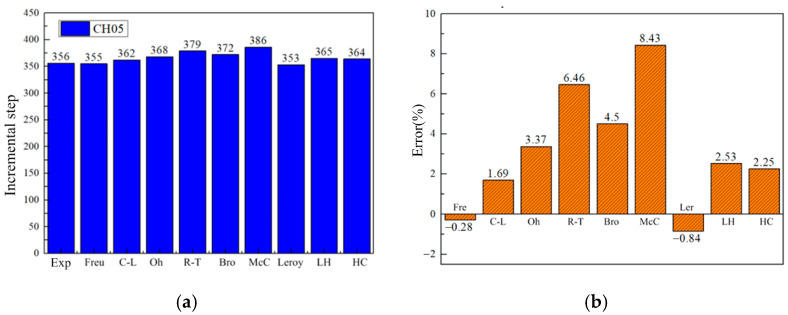
Damage initiation time for the CH05 sample under different criteria. (**a**) The corresponding incremental steps of damage appearing. (**b**) Damage error distribution.

Figure 23 shows the crack initiation time and damage error distribution for the CH05 sample under different criteria. It can be seen that the damage criteria still have a strong ability to predict damage in the CH05 sample, and all prediction errors are less than 10%.

**Figure 24:**
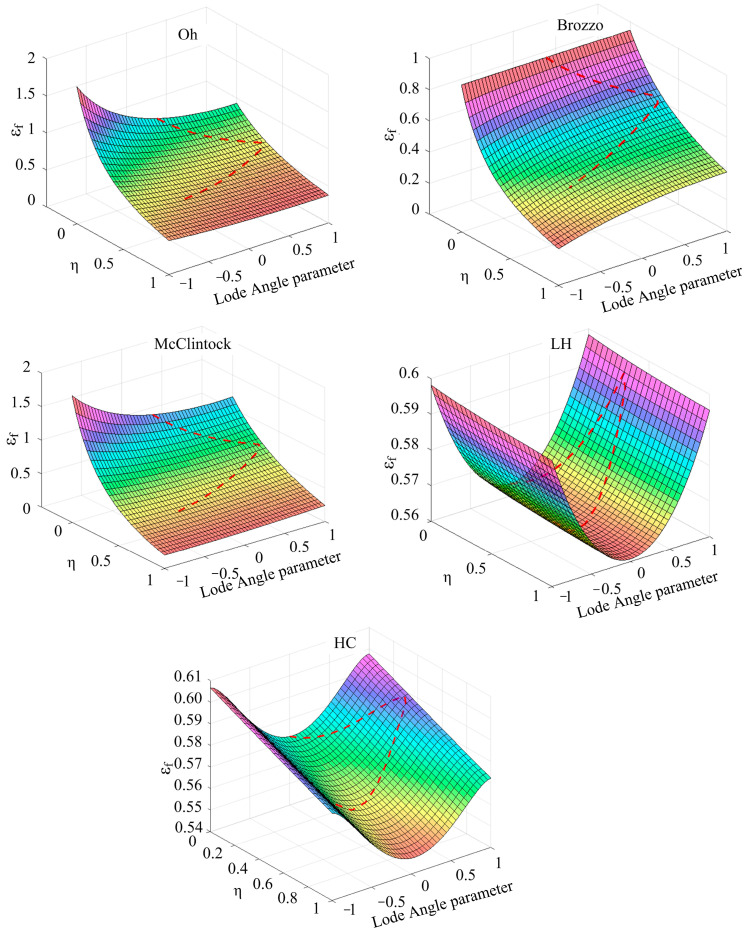
Fracture trajectories under different criteria.

In the above fracture criteria, the fracture trajectory considering the Lode angle parameter is shown in Figure 24. It can be seen from the Oh model that under the condition of low stress triaxiality and a low Lode angle parameter, the required fracture strain value is higher. Under the condition of low stress triaxiality, the variation in the Lode angle parameter has a great influence on fracture strain. Under the condition of a low Lode angle parameter, the change in stress triaxiality has a great influence on the fracture strain. According to the Brozzo criterion, it can be seen that under the condition of a high Lode angle parameter and low stress triaxiality, the fracture strain value is relatively high. Under high stress triaxiality, the fracture strain presents a nonlinear change with the increase in the Lode angle parameter, and when the stress triaxiality transitions to a low value, the fracture strain gradually changes from a nonlinear change to a linear change. The change trend in the McC criterion is similar to that in the Oh criterion. According to the L-H criterion, when the stress triaxiality is constant, the fracture strain presents a trend of decreasing first and then increasing with the increase in the Lode angle parameter. The fracture strain distribution of the H-C criterion is similar to that of the L-H criterion.

## 5. Conclusions

(1) Four types of samples were designed for SUS304 stainless steel, uniaxial tensile tests were carried out, and corresponding finite element models were established. The simulation results are compared with the test results, and the error is small. At the same time, DIC technology is used to measure the strain field of the DB sample, and the strain measurement results are compared with the simulation results. Both methods verify the accuracy of the simulation model.
(2) Based on the finite element software MSC.MARC, the HYPELA2 subroutine was written and the universal ductile fracture criterion was established. The feasibility of the HYPELA2 subroutine is verified by comparing it with the PLOTV subroutine and the UDAMAGE_INDICATOR subroutine, taking the three-point bending simulation as an example.
(3) A variety of ductile fracture criteria were calibrated according to the four tensile samples, and the error analysis was carried out according to the calibration results. The results show that the Freudenthal, C-L, and LeRoy criteria have a better predictive ability for the SH sample, while the error of the other criteria is relatively large. NT20 and CH05 samples are suitable for all criteria.

## Figures and Tables

**Table 1:** Ductile fracture criterion formula.

Damage Criterion	Formula
Freudenthal	$\int_{0}^{{\bar{\varepsilon }}_{f}^{p}}\sigma_{e}d{\bar{\varepsilon }}^{p}=C_{1}$
C-L	$\int_{0}^{{\bar{\varepsilon }}_{f}^{p}}\sigma_{1}d{\bar{\varepsilon }}^{p}=C_{2}$
Oh	$\int_{0}^{{\bar{\varepsilon }}_{f}^{p}}\frac{\sigma_{1}}{\sigma_{e}}d{\bar{\varepsilon }}^{p}=C_{3}$
R-T	$\int_{0}^{{\bar{\varepsilon }}_{f}^{p}}\exp(\frac{3\sigma_{m}}{2\sigma_{e}})d{\bar{\varepsilon }}^{p}=C_{4}$
Brozzo	$\int_{0}^{{\bar{\varepsilon }}_{f}^{p}}\frac{2\sigma_{1}}{3(\sigma_{1}-\sigma_{m})}d{\bar{\varepsilon }}^{p}=C_{5}$
McC	$\int_{0}^{{\bar{\varepsilon }}_{f}^{p}}\left[\frac{\sqrt{3}}{2(1-n)}\sin h\{\frac{\sqrt{3}}{2(1-n)}\frac{\sigma_{1}+\sigma_{2}}{\sigma_{e}}\}+\frac{3}{4}\frac{\sigma_{1}-\sigma_{2}}{\sigma_{e}}\right]d{\bar{\varepsilon }}^{p}=C_{6}$
LeRoy	$\int_{0}^{{\bar{\varepsilon }}_{f}^{p}}\left(\sigma_{1}-\sigma_{m}\right)d{\bar{\varepsilon }}^{p}=C_{7}$
L-H	$\int_{0}^{{\bar{\varepsilon }}_{f}^{p}}\frac{(f_{1}-f_{3}{)}^{C_{8}}(\left\langle 1+3\eta \right\rangle /2{)}^{C_{9}}}{C_{10}}d{\bar{\varepsilon }}^{p}=1,\;\;\left\langle 1+3\eta \right\rangle =\left\{\begin{array}{c} 0\;\;\;\;\;\;1+3\eta <0\\ 1+3\eta \;\;\;1+3\eta \geq 0 \end{array}\right.$
H-C	$\begin{array}{c} \int_{0}^{{\bar{\varepsilon }}_{f}^{p}}\frac{1}{b(1+c{)}^{\frac{1}{n}}g(\eta ,\bar{\theta })}d{\bar{\varepsilon }}^{p}=1,\\ g(\eta ,\bar{\theta })=\{(\frac{1}{2}((f_{1}-f_{2}{)}^{a}+(f_{2}-f_{3}{)}^{a}+(f_{1}-f_{3}{)}^{a}){)}^{\frac{1}{a}}+c(2\eta +f_{1}+f_{3}){\}}^{-\frac{1}{n}} \end{array}$

**Table 2:** Calibration results for different criteria.

Ductile Fracture Criterion	Parameters
Freudenthal	*C*_1_ = 782.64
C-L	*C*_2_ = 850.55
Oh	*C*_3_ = 0.62
R-T	*C*_4_ = 1.09
Brozzo	*C*_5_ = 0.63
McClintock	*C*_6_ = 1.25
LeRoy	*C*_7_ = 512.53
L-H	*C*_8_ = 0.4163, *C*_9_ = 0.0035, *C*_10_ = 0.5968
H-C	*a* = 1.8944, *b* = 0.5891, *c* = 0.0022, *n* = 0.1

## Data Availability

The original contributions presented in this study are included in the article. Further inquiries can be directed to the corresponding author.
